# Dynamic Leaf Physiology and Architecture Shape Fusarium Head Blight Resistance in Wheat

**DOI:** 10.3390/plants15010085

**Published:** 2025-12-27

**Authors:** Valentina Spanic, Jurica Duvnjak, Katarina Sunic Budimir, Josip Haramija, Andrea Ghirardo, Jörg-Peter Schnitzler

**Affiliations:** 1 Department of Small Cereal Crops Breeding and Genetics, Agricultural Institute Osijek, Juzno Predgradje 17, 31000 Osijek, Croatia; jurica.duvnjak@poljinos.hr (J.D.); katarina.sunic@poljinos.hr (K.S.B.); 2 Croatian Society of Agronomists, Berislavićeva 6/1, 10000 Zagreb, Croatia; josip.haramija1@gmail.com; 3 Helmholtz Munich, Research Unit Environmental Simulation, Ingolstädter Landstrasse 1, 85764 Neuherberg, Germany; andrea.ghirardo@helmholtz-munich.de (A.G.); joergpeter.schnitzler@helmholtz-munich.de (J.-P.S.)

**Keywords:** Fusarium head blight, leaf physiology, leaf morphology

## Abstract

Fusarium head blight (FHB) severely impacts wheat yield and grain quality, threatening global food security. In a field experiment, key photosynthetic, water relations, and leaf angular (morphological) traits were measured in the flag leaves of FHB-resistant and FHB-susceptible wheat genotypes under *Fusarium*-inoculated conditions. Measurements were conducted at 10 and 18 days post-inoculation (dpi) to evaluate the genotype- and time-dependent physiological and structural responses of resistant vs. susceptible genotypes to FHB infection over time. *Fusarium* infection induced distinct time- and genotype-specific changes across multiple physiological traits. At 10 dpi, when no visible symptoms were observed in either genotype, the resistant variety exhibited increased stomatal and total conductance, enhanced transpiration, earlier reductions in vapor pressure and H_2_O mole fractions, improved photosynthetic efficiency, and dynamic leaf pitch adjustments, while the susceptible variety decreased them. By 18 dpi, the resistant genotype had recovered water vapor dynamics and reversed leaf pitch changes, whereas the susceptible variety continued to exhibit physiological disruption. These results are consistent with the possibility that the coordinated regulation of water vapor conductance, leaf water status, photosynthetic performance, and leaf orientation contributes to FHB resistance. Understanding the interplay between physiological and morphological traits at early infection could guide targeted breeding strategies and early phenotypic selection tools.

## 1. Introduction

Wheat (*Triticum aestivum* L.) is a staple crop that provides approximately 20% of the calories in the human diet [1]. Climate change is expected to increase drought frequency and change rainfall intensities, leading to significant declines in food production. For example, wheat yields are projected to decrease substantially under future climate scenarios, with studies estimating yield reductions in the range of roughly 30–50% by mid-century due to combined effects of increased temperature, drought stress, and altered precipitation patterns, depending on the region and management practices [2]. Meeting the rising global demand for wheat is increasingly challenging due to limitations such as reduced arable land, climate change, abiotic stresses, and the significant impact of pathogenic fungal infections on grain yield and quality [3]. Fusarium head blight (FHB), primarily caused by *Fusarium graminearum* Schwabe and *F. culmorum* W.G. Smith, is a major fungal disease affecting wheat production worldwide, posing significant economic and food security challenges [4]. *Fusarium* infection negatively impacts grain yield by triggering physiological changes in the host plant, notably by impairing photosynthesis [5]. The flag leaf, the last and uppermost leaf in wheat, plays a critical role during grain filling by supplying most of the photosynthates that support grain development. The developing grains act as sink tissues, relying on the imported carbohydrates for growth and storage [6,7]. Efficient photosynthesis in the flag leaf strongly correlates with higher grain yield and quality [8]. Enhancing photosynthetic efficiency represents a major goal for increasing crop productivity and ensuring global food security. Field phenotyping plays a vital role in elucidating the physiological and environmental constraints that define crop performance under agricultural conditions [9].

Sunic et al. [10] reported that enhanced ascorbate–glutathione (AsA–GSH) metabolism in FHB-resistant varieties maintained spike redox balance and photosystem II (PSII) functionality. In contrast, carotenoids, pigments essential for PSII assembly and photoprotection, declined under FHB stress in both resistant and moderately resistant varieties. In another study, it was seen that at one testing location, earlier declines in the maximum quantum yield of primary photochemistry (TR_0_/ABS) and performance index on an absorption basis (PI_abs_) in glumes and flag leaves of wheat due to increased FHB pressure suggest that reduced photosynthetic efficiency shortened the grain-filling period and caused spikelet sterility through impaired pollination [11]. Barley yellow dwarf virus infections significantly reduced ^14^CO_2_ assimilation by flag leaves and the export of photosynthates to wheat spikes in both varieties, with the effect being more pronounced in the moderately FHB-resistant variety compared to the FHB-susceptible variety [12]. The study by Wagner et al. [13] demonstrated that cover crops influence the pathogenicity of *F. oxysporum* populations and revealed a correlation between disease severity and reduced photosynthetic efficiency in tomato plants. Also, it was reported that the second line of defense triggered by *F. graminearum* infection involves a moderated suppression of photosynthesis and the activation of genes responsive to biotic stress [14]. Gene expression analysis revealed an overall inhibition of photosynthesis in wheat genotypes following *F. graminearum* infection. This reduction is likely a consequence of host-driven resource reallocation toward defense processes, consistent with the growth–defense trade-off concept, rather than a direct suppression of photosynthesis by the pathogen [15].

Although direct reports of *Fusarium*-induced changes in leaf vapor pressure per se are scarce, analogous work has shown that *F. graminearum* infection perturbs stomatal conductance and leaf hydraulics [16,17], both of which are closely linked to leaf vapor pressure dynamics. Notably, in abiotic contexts, the regulation of transpiration in response to increasing the vapor pressure deficit has been shown to influence water-use efficiency and crop performance in durum wheat [18]. Recent drought studies showed that reductions in stomatal conductance and leaf hydraulic conductance are tightly linked to decreases in leaf water potential, highlighting a shift in stomatal behavior as soil moisture declines [19]. Leaf hydraulic conductance plays a central role in regulating stomatal response and water vapor dynamics, yet its interplay with pathogen-induced stress, such as FHB, remains largely unexplored. Given that both hydraulic conductance and leaf orientation modulate transpiration and gas exchange, understanding their combined effects under biotic stress is crucial. Leaf angle influences the microenvironment around the leaf by affecting boundary layer thickness and vapor exchange, which in turn impact stomatal conductance (g_s_) and vapor pressure gradients. Specifically, VP_leaf_ represents the vapor pressure at the leaf surface, while VPD_leaf_ (vapor pressure deficit) describes the difference between leaf and ambient vapor pressures, determining the driving force for transpiration. For example, when leaves are more vertical, evaporation may be reduced or vapor dispersion may be changed, which can modify water vapor conductance [20]. Further, more erect (steeper) leaf angles reduce the exposed surface area facing excess sun/heat, and thus can reduce leaf temperature, transpiration, and water loss; conversely, more horizontal leaves may increase interception but also increase water loss [21]. Leaf angle and architecture also influence how leaves intercept moisture (rain, fog) or channel water toward roots (especially in arid/drought conditions), which is related to vapor pressure/mole fraction traits because water uptake, leaf hydraulics, and water vapor dynamics are all part of the same system [22]. Despite growing evidence of their role in water-use regulation, the involvement of leaf angular traits in plant responses to *Fusarium* infection and their relationship with vapor pressure dynamics has yet to be fully investigated.

However, the physiological and morphological responses, particularly those related to gas exchange, photosynthesis, leaf vapor pressure dynamics, and angular leaf orientation, remain less understood in FHB-resistant and FHB-susceptible wheat genotypes under *Fusarium* infection. Therefore, this study aimed to investigate the effects of *Fusarium* spp. infection on stomatal conductance and transpiration rates, photosynthetic performance, vapor pressure dynamics, and leaf angular traits (e.g., pitch, azimuth, and roll) in two wheat varieties differing in FHB resistance, Vulkan (resistant) and Golubica (susceptible), at 10 and 18 dpi.

## 2. Results

Measurements of stomatal conductance and transpiration rates, as well as photosynthetic, vapor pressure-related, and leaf angular traits, were conducted on FHB-resistant (Vulkan) varieties and compared to FHB-susceptible (Golubica) genotypes at 10 and 18 days post-inoculation (dpi) with *Fusarium* spp.

### 2.1. Disease Assessment

At 10 dpi, no visible infection symptoms were observed in either wheat varieties (Table 1). At 18 dpi, symptoms developed only in the susceptible genotype Golubica, while the resistant genotype Vulkan remained symptom-free for Type I resistance (score = 0) and showed minimal general resistance symptoms (score = 2.5). Disease severity, expressed as the area under the disease progress curve (AUDPC), was much higher in Golubica (general = 271.3; Type I = 175) than in Vulkan (general = 21.1; Type I = 11.8). No symptoms occurred in the control plots.

### 2.2. Stomatal Conductance and Transpiration Rates

**Figure 1:**
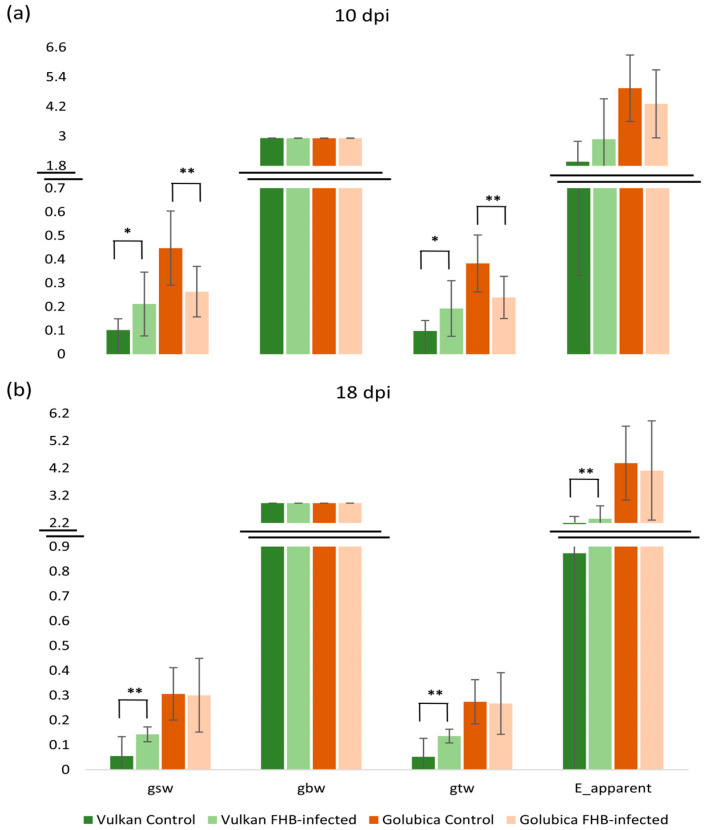
Stomatal conductance to water vapor (gsw), one-sided boundary layer conductance (gbw), total conductance to water vapor (gtw), and transpiration (E_apparent) of the Vulkan (FHB-resistant) and Golubica (FHB-susceptible) varieties (**a**) at 10 days post-inoculation (dpi) and (**b**) at 18 dpi after two treatments (control and FHB-infected). Asterisks indicate statistically significant differences (* *p* < 0.05, ** *p* < 0.01, two-sample *t*-test) between treatments within each variety. Bars represent mean values ± SD (n = 10). Gsw, gbw, and gtw are expressed as mol m^−2^ s^−1^, while E_apparent is expressed as mmol m^−2^ s^−1^.

At 10 dpi, stomatal conductance to water vapor (gsw), which reflects the rate of gas diffusion through open stomata, and total conductance to water vapor (gtw), representing the combined stomatal and boundary layer conductance, were significantly higher in inoculated plants of the FHB-resistant variety, Vulkan, than in the non-inoculated control (Figure 1a). In contrast, the FHB-susceptible Golubica variety exhibited a significant decrease in both gsw and gtw following inoculation. At 18 dpi, this trend persisted but was only significant in the resistant variety (Figure 1b). In the FHB-resistant plants, gsw, gtw, and the apparent transpiration rate (E_apparent) remained significantly higher in inoculated plants relative to their respective controls. One-layer boundary conductance (gbw) remained unchanged in response to FHB infection at both measurement points.

### 2.3. Vapor Pressure-Related Traits

**Figure 2:**
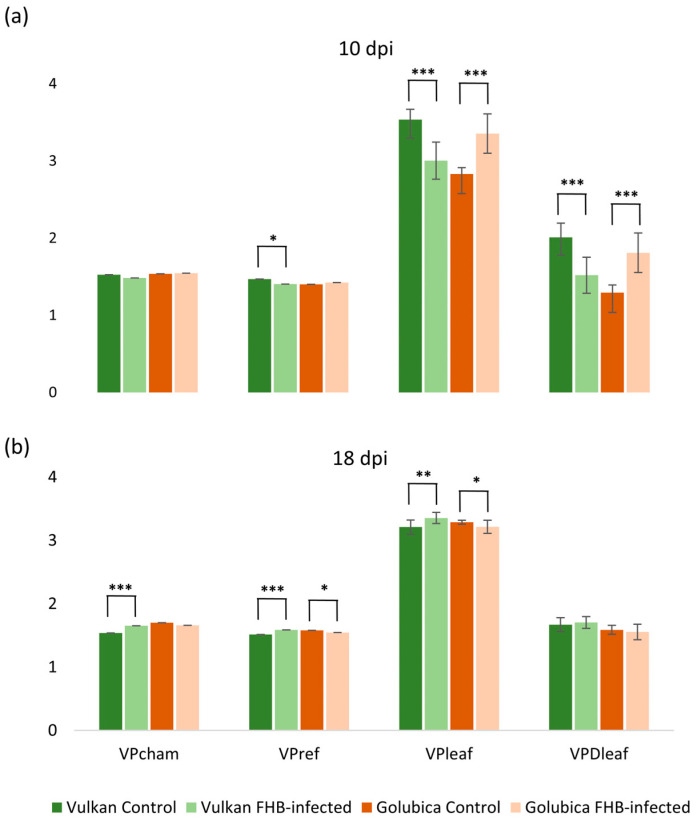
Chamber vapor pressure (VP_cham_), reference vapor pressure (VP_ref_), leaf vapor pressure (VP_leaf_), and vapor pressure deficit (VPD_leaf_) of the Vulkan (FHB-resistant) and Golubica (FHB-susceptible) varieties (**a**) at 10 days post-inoculation (dpi) and (**b**) at 18 dpi after two treatments (control and FHB-infected). Asterisks indicate statistically significant differences (* *p* < 0.05, ** *p* < 0.01, *** *p* < 0.001, two-sample *t*-test) between treatments within each variety. Bars represent mean values ± SD (n = 10). VP_cham_, VP_ref_, VP_leaf_, and VPD_leaf_ are expressed as kPa.

At 10 dpi, significant differences were observed in vapor pressure-related traits between the resistant and susceptible wheat varieties under *Fusarium* treatment. In the resistant variety, *Fusarium* infection caused a significant reduction in reference vapor pressure (VP_ref_), which represents the ambient air vapor pressure, as well as in leaf vapor pressure (VP_leaf_), indicating lower moisture on the leaf surface. Consequently, the vapor pressure deficit (VPD_leaf_), the gradient driving water vapor loss from the leaf to the atmosphere, was also reduced compared to the non-inoculated control (Figure 2a). In contrast, the susceptible variety exhibited a significant increase in both VP_leaf_ and VPD_leaf_ under *Fusarium* treatment, while VP_cham_ and VP_ref_ remained unchanged. These results suggest differential physiological responses to *Fusarium* infection between the two genotypes, particularly in traits related to leaf water vapor dynamics. At 18 dpi, the resistant variety exhibited a significant increase in chamber vapor pressure (VP_cham_), VP_ref_, and VP_leaf_ under *Fusarium* treatment (Figure 2b).

**Figure 3:**
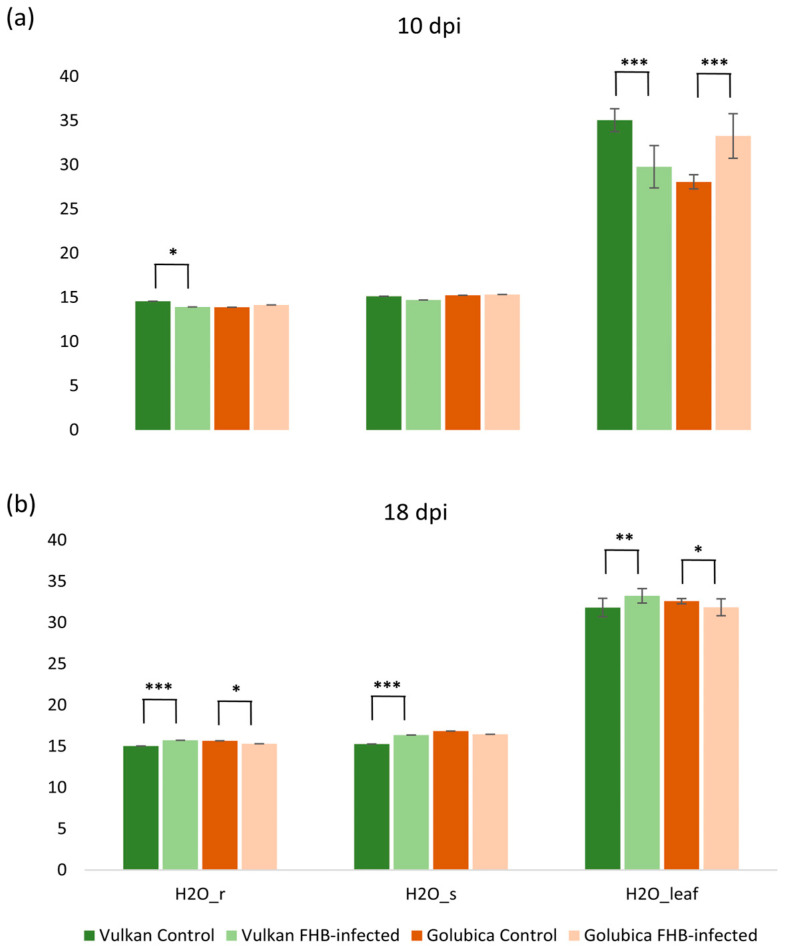
Reference H_2_O mole fraction (H_2_O_r_), chamber H_2_O mole fraction (H_2_O_s_), and leaf H_2_O mole fraction (H_2_O_leaf_) of the Vulkan (FHB-resistant) and Golubica (FHB-susceptible) varieties (**a**) at 10 days post-inoculation (dpi) and (**b**) at 18 dpi after two treatments (control and FHB-infected). Asterisks indicate statistically significant differences (* *p* < 0.05, ** *p* < 0.01, *** *p* < 0.001, two-sample *t*-test) between treatments within each variety. Bars represent mean values ± SD (n = 10). H_2_O_r_, H_2_O_s_, and H_2_O_leaf_ are expressed as mmol mol^−1^.

Conversely, in the susceptible variety, both VP_ref_ and VP_leaf_ were significantly decreased compared to the control, indicating a continued disruption of leaf water vapor dynamics. These contrasting responses highlight a differential temporal regulation of vapor pressure traits between resistant and susceptible genotypes in response to *Fusarium* infection. At 10 dpi, *Fusarium* infection led to a significant decrease in the reference H_2_O mole fraction (H_2_O_r_) and leaf H_2_O mole fraction (H_2_O_leaf_) in the resistant variety compared to the control (Figure 3a). In contrast, the susceptible variety showed a significant increase in H_2_O_leaf_ under *Fusarium* treatment, while H_2_O_r_ and H_2_O_s_ remained unchanged. At 18 dpi, the resistant variety exhibited a significant increase in H_2_O_r_, chamber H_2_O mole fraction (H_2_O_s_), and H_2_O_leaf_ under *Fusarium* treatment, suggesting a recovery or compensatory response in water vapor dynamics (Figure 3b). Conversely, in the susceptible variety, both H_2_O_r_ and H_2_O_leaf_ were significantly decreased compared to the control, indicating continued disruption of water vapor balance under infection.

### 2.4. FHB-Induced Alterations in PSII Efficiency and Electron Transport Activity

**Figure 4:**
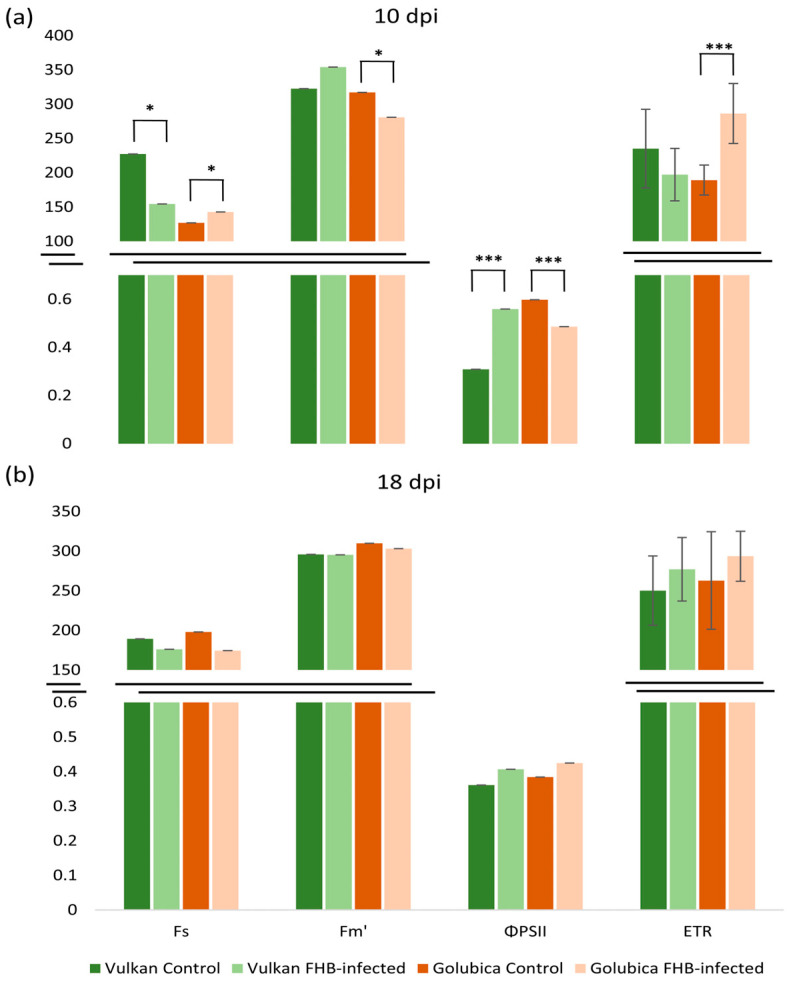
Minimum fluorescence in light (F_s_), maximum fluorescence in light (F_m_′), quantum efficiency of PSII in light (ΦPSII), and electron transport rate (ETR) of the Vulkan (FHB-resistant) and Golubica (FHB-susceptible) varieties (**a**) at 10 days post-inoculation (dpi) and (**b**) at 18 dpi after two treatments (control and FHB-infected). Asterisks indicate statistically significant differences (* *p* < 0.05, *** *p* < 0.001, two-sample *t*-test) between treatments within each variety. Bars represent mean values ± SD (n = 10). ETR is expressed as µmol of electrons m^−2^ s^−1^.

At 10 dpi, the FHB-resistant Vulkan variety showed a significant decrease in minimum fluorescence in light (F_s_) and a significant increase in quantum efficiency of PSII in light (ΦPSII) under FHB treatment compared to the control (Figure 4a). In contrast, the susceptible variety exhibited a significant increase in F_s_ and a significant decrease in ΦPSII, along with an increase in electron transport rate (ETR). Additionally, the susceptible variety showed a significant decrease in maximum fluorescence in light (F_m_′) under FHB treatment. At 18 dpi, none of the measured fluorescence parameters showed significant differences between treatments (Figure 4b).

### 2.5. Leaf Angular Traits

**Figure 5:**
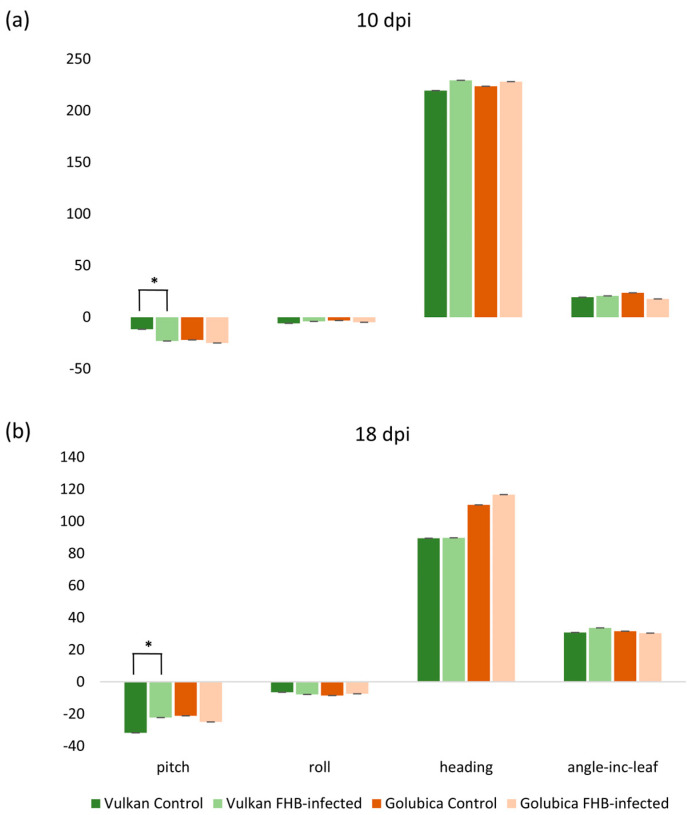
Slope from horizontal (pitch), rotation from horizontal (roll), rotation from north (heading) and angle of incidence (angle_Inc_leaf) between the leaf and the sun in the Vulkan (FHB-resistant) and Golubica (FHB-susceptible) varieties (**a**) at 10 days post-inoculation (dpi) and (**b**) at 18 dpi after two treatments (control and FHB-infected). Asterisks indicate statistically significant differences (* *p* < 0.05, two-sample *t*-test) between treatments within each variety. Bars represent mean values ± SD (n = 10). Pitch, roll, heading, and angle_Inc_leaf are expressed as degrees.

The pitch was significantly increased in the FHB-resistant Vulkan variety under *Fusarium* treatment compared to the control at 10 dpi (Figure 5a). However, at 18 dpi, pitch was significantly decreased under *Fusarium* treatment (Figure 5b). In contrast, the susceptible variety showed no significant changes in pitch (slope from horizontal) or any other orientation traits (roll-rotation from horizontal, heading-rotation from north, angle_Inc_leaf-angle of incidence between the leaf and the sun) at either time point. All other traits were non-significantly affected in both varieties across all treatments and time points.

## 3. Discussion

This present study shows that FHB infection significantly alters photosynthetic performance in wheat, with resistant and susceptible genotypes exhibiting distinct physiological responses during early infection. Resistant genotypes maintained higher stomatal conductance, total conductance, and transpiration, whereas susceptible genotypes showed reduced or unchanged values, likely reflecting stomatal closure [5]. Photosystem II, a key site of reactive oxygen species generation, plays an important role in early defense responses [14]. Previous studies reported that *F. graminearum* infection disrupts photosynthesis, with resistant and susceptible genotypes differing in net photosynthesis, and additional leaf damage may further affect source–sink balance [23,24]. Our results indicate that both Type I resistance and general resistance responses may display similar physiological patterns during early infection [25]. By integrating structural traits such as leaf angle with functional traits like hydraulic conductance and gas exchange, this study provides a comprehensive understanding of how FHB influences the coordination between photosynthesis and transpiration under disease stress.

### 3.1. Stomatal Conductance and Transpiration Parameters in Flag Leaves Under Fusarium Stress

Photosynthesis and water regulation are tightly linked processes that are widely considered to determine plant performance under environmental stress, including pathogen attack. Responses of stomatal conductance (gsw), transpiration rate, and net photosynthetic rate to environmental factors are key to evaluating evapotranspiration and ecosystem productivity in agroecosystems [26]. At 10 dpi, the FHB-resistant wheat variety showed increases in stomatal conductance (gsw), total conductance (gtw), and apparent transpiration (E_apparent), which may indicate that stomata remained open and facilitated greater water and CO_2_ exchange. Conversely, in the susceptible variety, these parameters declined or showed limited change, which may be associated with stomatal closure triggered by infection, and is consistent with observed disease symptoms. In resistant plants, *Fusarium* inoculation does not appear to result in extensive infection, which may allow stomata to remain open and gas exchange and leaf cooling to be maintained, thereby potentially mitigating early stress. Francesconi and Balestra [16] found that the expressions of genes that promote stomatal closure was upregulated in the FHB resistant variety “Sumai 3” after *F. graminearum* inoculation, whereas in the FHB-susceptible variety, those responses were weaker, suggesting that defense mechanisms in resistant varieties may involve stomatal regulation. Another study, comparing FHB-resistant vs. susceptible genotypes, observed that infection reduced the net photosynthetic rate and stomatal conductance of flag leaves, and that the magnitude of effect was greater in the resistant genotype. Specifically, resistant plants tended to show decreases in photosynthesis and stomatal conductance after FHB infection, but susceptible genotypes showed a greater reduction of yield components [27]. In FHB-susceptible varieties, infection is likely to impose stress (toxicity, damage, and water stress) that leads stomata to close or reduce conductance, both as a passive result (damage) or active response to reduce water loss or pathogen spread. Ref. [28] showed that *Fusarium* infection significantly reduced stomatal conductance in leaves of wheat seedlings, which was interpreted as reflecting impaired water regulation caused by vascular damage and water stress. Previous work has also shown that photosynthetic and stomatal responses can vary between early (10 dpi) and later (26 dpi) stages of infection. In flag leaves and spikes of winter wheat, parameters related to photochemistry (chlorophyll fluorescence) have been observed to respond at early phases of infection and again around 18 dpi [29]. After a somewhat longer infection period (18 dpi) in this present study, FHB-resistant plants appeared to maintain or partially recover leaf function, with sustained stomatal opening and transpiration that may support photosynthesis or cooling. In contrast, in FHB-susceptible plants, greater damage or loss of function was evident, and these plants were less able to maintain comparable rates. In line with our findings, pre- and post-anthesis photosynthetic traits, such as net CO_2_ assimilation under varying light intensities, have been shown to correlate positively with grain yield and harvest index [9], highlighting the importance of maintaining photosynthetic efficiency under FHB stress.

### 3.2. Impact of Fusarium Infection on Vapor Pressure Dynamics in Wheat Flag Leaves

We also examined how *Fusarium* infection is associated with changes in vapor pressure-related traits in the flag leaves of resistant and susceptible wheat genotypes over time. The contrasting responses between genotypes and the temporal dynamics (10 dpi vs. 18 dpi) suggest complex interactions between pathogen damage, plant hydraulic regulation, and stomatal behavior. At 10 dpi, the resistant variety exhibited reduced VP_ref_, VP_leaf_, and VPD_leaf_ compared to the control, which may reflect tighter stomatal or hydraulic regulation that could help limit pathogen spread. In contrast, the susceptible variety showed increased VP_leaf_ and VPD_leaf_, which may indicate a loss of water control early in infection. Such pathogen-induced disruption of stomatal regulation is consistent with previous reports that pathogens can impair guard cell function or disrupt stomatal opening/closing (e.g., by toxin effects or hormonal interference) and thereby alter plant water relations [30]. In wheat, resistance to FHB has been associated with the upregulation of stomatal-closure genes, supporting the hypothesis that active stomatal control may contribute to disease resistance [16]. At 18 dpi, the resistant genotype displayed a significant increase of VP_cham_, VP_ref_, and VP_leaf_, which may suggest recovery or compensation of vapor pressure dynamics. Meanwhile, the susceptible variety exhibited significant declines, reflecting progressive tissue or vascular damage that could impair water transport [31]. We observed significant, genotype-specific, and time-dependent changes in reference (H_2_O_r_), chamber (H_2_O_s_), and leaf (H_2_O_leaf_) vapor mole fractions in flag leaves of two wheat varieties differing in FHB resistance. At 10 dpi, *Fusarium* infection was associated with a significant decrease in both H_2_O_r_ and H_2_O_leaf_ in the resistant variety, which may indicate an early stomatal or hydraulic regulation to limit pathogen spread [32]. In contrast, the susceptible variety showed a significant increase in H_2_O_leaf_, indicating failure to restrict water vapor exchange, likely due to impaired stomatal control or increased apoplastic water. These contrasting responses support a growth–defense trade-off, where resistant plants temporarily reduce gas exchange to prioritize survival [15]. At 18 dpi, the resistant variety showed a significant increase in H_2_O_r_, H_2_O_s_, and H_2_O_leaf_, indicating recovery or compensation in water vapor dynamics. In contrast, the susceptible variety showed declines, reflecting progressive tissue degradation, impaired water transport, and the breakdown of stomatal and vascular integrity. Vascular blockage reduces leaf water supply and collapses transpiration-driven vapor gradients, explaining the decline in H_2_O mole fractions in the susceptible genotype [31].

### 3.3. Impact of FHB Infection on PSII Efficiency and Electron Transport

In this study, we observed that at 10 days post-inoculation (dpi), the FHB-resistant variety and FHB-susceptible variety differed in their chlorophyll fluorescence and photosynthetic parameters under FHB treatment, but at 18 dpi, these differences were not detected. Together, these observations suggest that in the resistant variety, early infection (10 dpi) may trigger adaptive or compensatory responses in the photosynthetic apparatus, such as reduced energy dissipation, the altered regulation of non-photochemical quenching (NPQ), or maintenance of reaction center efficiency under light, potentially through enhanced antioxidant protection or repair mechanisms [33]. Sunic et al. [10] found that in winter wheat, resistant varieties under FHB stress tended to maintain photosystem II functionality more effectively, in part via stronger AsA–GSH antioxidant responses that may help protect the redox state of spikes and contribute to preserving PSII function. In contrast, this present study indicates that in the susceptible variety, these changes are consistent with stress effects on PSII, such as a reduction in the proportion of open reaction centers, limitations in downstream electron transport, or partial damage or inactivation of photosynthetic components. This suggests that susceptible varieties may experience functional impairment of PSII during the early stages of infection. Our findings are consistent with previous research showing that photosynthetic parameters are particularly informative during the early stages of *Fusarium* infection. For example, Ajigboye et al. [34] demonstrated that ET_0_/RC and F_v_′/F_m_′ can serve as sensitive indicators of *Fusarium*-induced stress, capable of distinguishing between resistant and susceptible wheat genotypes early in the infection phase. This corresponds with our observations at 10 dpi, where significant differences in PSII efficiency and electron transport were observed between the resistant and susceptible varieties.

### 3.4. Morphological Response to Fusarium Infection

Little is known about whether leaf orientation changes in wheat are actively regulated in response to biotic stress, particularly in the flag leaf, and whether these responses differ between FHB-resistant and susceptible genotypes. In studies of leaf motion, small changes in pitch and roll have been shown to allow plants to react to external stresses, and IMU sensors can detect subtle leaf movement under stress [35]. The results of this present study show a transient alteration of pitch in the FHB-resistant wheat variety in response to *Fusarium* treatment, whereas the FHB-susceptible variety did not exhibit significant changes in leaf orientation traits. This observation suggests that pitch modulation may be associated with resistance-associated responses to fungal stress. Similar stress-induced leaf reorientation has been reported in drought studies, where flag leaf angles were altered to mitigate stress effects [22,36]. The significant increase in pitch at 10 dpi in the resistant variety may reflect a rapid adjustment in leaf water status, which could contribute to maintaining transpiration and cooling while potentially reducing the impact of early *Fusarium* infection. The FHB-susceptible variety did not show a significant response, which may indicate a reduced capacity for orientation-based morphological plasticity under pathogen stress. Previous studies have shown that leaf wettability and leaf angle can influence air-moisture deposition in wheat, enhancing self-irrigation and helping the plant maintain water status under stress [37]. In addition, leaf rolling has been reported as an early adaptive response that can precede stomatal closure under drought in rice, reducing water loss and helping maintain leaf water status [38].

## 4. Materials and Methods

### 4.1. Experimental Layout

**Figure 6:**
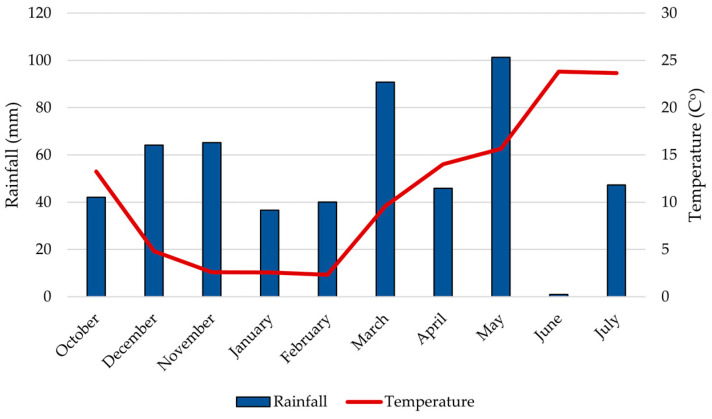
Total monthly rainfall (mm) and average temperature (°C) during the growing season from October 2024 to June 2025 in Osijek.

Field research was conducted during the 2024–2025 growing season at the Agricultural Institute Osijek, Croatia (45°27′ N, 18°48′ E), on eutric cambisol soil (pHKCl–6.25; humus 2.00–2.20%). The experiment focused on two winter wheat varieties developed by the Agricultural Institute Osijek: Vulkan (FHB-resistant) and Golubica (FHB-susceptible) [11,24]. The experimental design was a randomized complete block with two treatments (*Fusarium*-inoculated and untreated control) and two replications. Each plot was 7 × 1.08 m (length × width). Seeds were sown using a Hege Seedmatic drill (Wintersteiger, Ried im Innkreis, Austria) in October 2024 at a density of 3000–3500 plants per 7.56 m^2^. To control seed-borne diseases, seeds were treated with MAXIM^®^ (Washington, DC, USA, Fludioxonil) at a rate of 125 mL per 100 kg of seed. Standard agro-technical practices for commercial wheat production were applied, except that no fungicides were used during the growing season. In February 2025, herbicide treatments included Quelex (Halauksifen-metil 104.2 g kg^−1^ + Florasulam 100 g kg^−1^, Corteva. Cremona, Italy). In March, further treatments were applied using Moddus 250 EC (trineksapak-etil 250 g L^−1^, Syngenta, Basel, Switzerland) at 0.33 L ha^−1^, and in May, Cythrin max (cipermetrin 500 g L^−1^, Arysta LifeScience Benelux SPRL, Seraing, Belgium) at 0,07 L ha^−1^. Climatic data during the 2024–2025 growing season were obtained from the in situ agrometeorological station PinovaMeteo (Pinova, Čakovec, Croatia). Total precipitation during the season was 534.5 mm, and the average annual temperature was 11.2 °C (Figure 6).

### 4.2. Fusarium Inoculum Preparation and Inoculation Procedure

Two *Fusarium* species were used for inoculum production: *F. graminearum* (PIO 31), previously isolated from winter wheat in eastern Croatia, and *F. culmorum* (IFA 104), obtained from IFA-Tulln, Tulln, Austria. Conidial inoculum was produced on a sterilized grain substrate consisting of a wheat and oat mixture (3:1, *v*/*v*). For each isolate, two 5 mm-diameter mycelial disks from actively growing cultures were transferred into glass jars containing the pre-soaked and autoclaved grain mixture. The jars were incubated at room temperature under diffused daylight and shaken daily for two weeks to promote aeration and uniform fungal growth. After the incubation period, macroconidia were harvested by washing the colonized grains with sterile water. The resulting spore suspensions were filtered and diluted to a final concentration of 1 × 10^5^ conidia mL^−1^, determined using a Bürker-Türk hemocytometer (Hecht Assistent, Sondheim vor der Rhön, Germany). Before field application, equal volumes of the two *Fusarium* sore suspensions were mixed to create a 50:50 inoculum, which was then diluted in 100 L of water and applied at 100 mL m^−2^ to simulate epidemic FHB conditions. Two treatments were included in the experiment: (1) a non-inoculated control grown under standard agronomic practices without fungicide or misting and (2) an inoculated treatment subjected to two inoculation events at anthesis (Zadoks growth stage 65) [39]. Inoculation was carried out across the entire plot area using a backpack sprayer. To promote infection, supplemental misting was applied periodically using a tractor-mounted sprayer to maintain adequate humidity for fungal development.

### 4.3. Disease Severity and Initial Infection

Disease progression was evaluated by assessing both general disease severity and Type I resistance to FHB, which reflects resistance to initial infection within the spike. Assessments were conducted at 10, 14, 18, 22, and 26 days post-inoculation (dpi). Disease severity (general resistance) was estimated visually as the percentage of bleached spikelets per plot using a linear scale from 0 to 100%. Type I resistance (disease incidence) was determined by calculating the percentage of infected ears in a randomly selected sample of 30 wheat heads per plot. All disease severity and incidence data were used to calculate the area under the disease progress curve (AUDPC), a standard and widely used metric for quantifying disease development over time according to the following formula [40]:

$$AUDPC=\sum_{i=1}^{n}\left\{\left[\frac{Yi+Yi-1}{2}\right]*(Xi-Xi-1)\right\}$$

### 4.4. Stomatal Conductance, Transpiration Rates, Vapor Pressure-Related Traits, Chlorophyll Fluorescence, and Angular Trait Measurements

Physiological measurements of flag leaves were conducted at 10 and 18 dpi using a Portable Photosynthesis System (LI-600, LI-COR Biosciences, Lincoln, NE, USA) [41]. The LI-600 quantifies transpiration (E) and stomatal conductance using an open flow-through system that measures airflow and the change in water vapor mole fraction between the inlet and outlet of the measurement chamber. It also integrates a porometer and modulated fluorometer, allowing for a non-destructive assessment of stomatal conductance to water vapor (g_sx_) and chlorophyll fluorescence parameters under light conditions, along with leaf angular orientation traits. Measurements were performed in ambient field conditions between 9:00 and 11:30 a.m. on fully expanded flag leaves from five representative plants per plot (two plots in total). To minimize variability, leaves were measured under similar light exposure and minimal wind conditions. The leaf clamp was gently positioned to avoid altering the natural leaf angle during measurements. Prior to data collection, the device was zeroed and calibrated according to the manufacturer’s instructions. The g_sx_ was determined based on differential humidity measurements before and after the leaf surface using dual RH sensors and flow rate data. Leaf temperature was measured with the built-in infrared thermometer. Chlorophyll fluorescence parameters were collected from light-adapted leaves using modulated light pulses under similar light conditions. The measurements were fully randomized and the following variables were recorded: steady-state fluorescence (F_s_), maximum fluorescence in light-adapted state (F_m_′), effective quantum yield of PSII (ΦPSII = (F_m_′ − F_s_)/F_m_′), electron transport rate (ETR), calculated from ΦPSII, incident PAR, and estimated leaf absorptance. In addition to physiological traits, the LI-600’s built-in accelerometer and magnetometer enabled the recording of leaf angular traits, including pitch (leaf slope from horizontal plane), roll (lateral curvature relative to horizontal), heading (azimuth or rotation from north), and angle of incidence (angle_inc_leaf), calculated as the angle between the leaf surface and the direction of incoming solar radiation and leaf roll (lateral curvature).

### 4.5. Statistical Analysis

The obtained data were analyzed by analysis of variance (ANOVA) using an appropriate statistical model in Statistica software, version 12.0 (StatSoft Inc., Tulsa, OK, USA). Parameters measured by LI-600 Portable Photosynthesis System are presented as the mean ± standard deviation of ten independent biological replicates. Differences among treatments within each variety were assessed using a two-sample *t*-test with significance set at *p* < 0.05.

## 5. Conclusions

*Fusarium* infection appeared to elicit distinct and time-dependent physiological responses in wheat varieties differing in resistance to FHB. Resistant varieties appear to maintain water regulation, photosynthetic efficiency, and leaf orientation more effectively than susceptible varieties, suggesting coordinated adaptive mechanisms that mitigate early pathogen stress. In contrast, susceptible varieties show sustained disruption of water vapor dynamics and photosynthetic function, with limited recovery over time. These contrasting patterns indicate that FHB resistance may involve the early regulation of stomatal conductance, water status, photosystem performance, and leaf orientation, collectively supporting resilience under pathogen pressure. Overall, this study provides insight into the physiological basis of FHB resistance and highlights traits that could inform breeding strategies for improved wheat resilience. Together, these contrasting temporal patterns highlight that FHB resistance involves an early and coordinated regulation of water vapor conductance, leaf water status, photosynthetic efficiency, and leaf orientation, enabling the resistant genotype to maintain physiological function under FHB stress.

## Figures and Tables

**Table 1:** General and Type I resistance (resistance to initial infection) in two winter wheat varieties (Vulkan and Golubica) as determined in terms of area under disease progress curve (AUDPC). The data represent the mean of two replicates.

General Resistance	Symptom Evaluation Score at 10 dpi*	Symptom Evaluation Score at 18 dpi*	AUDPC
Vulkan	0	2.5	21.25
Golubica	0	27.5	271.25
Type I resistance			AUDPC
Vulkan	0	0	11.75
Golubica	0	17.5	175

* Disease symptoms were evaluated at 10 and 18 dpi using a 0–100 scale, with higher scores corresponding to greater disease severity.

## Data Availability

Data are contained within the article.
